# Photoacoustic microscopy for real-time monitoring of near-infrared optical absorbers inside biological tissue

**DOI:** 10.1117/1.JBO.29.S1.S11527

**Published:** 2024-03-09

**Authors:** Takeshi Hirasawa, Kazuyoshi Tachi, Tomohiro Ishikawa, Manami Miyashita, Keiichi Ito, Miya Ishihara

**Affiliations:** a National Defense Medical College, Department of Medical Engineering, Tokorozawa, Japan; b National Defense Medical College, Department of Urology, Tokorozawa, Japan

**Keywords:** photoacoustic, multispectral, real-time, microscopy, circulating tumor cell

## Abstract

**Significance:**

We developed a high-speed optical-resolution photoacoustic microscopy (OR-PAM) system using a high-repetition-rate supercontinuum (SC) light source and a two-axes Galvano scanner. The OR-PAM system enabled real-time imaging of optical absorbers inside biological tissues with excellent excitation wavelength tunability.

**Aim:**

In the near-infrared (NIR) wavelength range, high-speed OR-PAM faces limitations due to the lack of wavelength-tunable light sources. Our study aimed to enable high-speed OR-PAM imaging of various optical absorbers, including NIR contrast agents, and validate the performance of high-speed OR-PAM in the detection of circulating tumor cells (CTCs).

**Approach:**

A high-repetition nanosecond pulsed SC light source was used for OR-PAM. The excitation wavelength was adjusted by bandpass filtering of broadband light pulses produced by an SC light source. Phantom and *in vivo* experiments were performed to detect tumor cells stained with an NIR contrast agent within flowing blood samples.

**Results:**

The newly developed high-speed OR-PAM successfully detected stained cells both in the phantom and *in vivo*. The phantom experiment confirmed the correlation between the tumor cell detection rate and tumor cell concentration in the blood sample.

**Conclusions:**

The high-speed OR-PAM effectively detected stained tumor cells. Combining high-speed OR-PAM with molecular probes that stain tumor cells *in vivo* enables *in vivo* CTC detection.

## 1 Introduction

Optical-resolution photoacoustic microscopy (OR-PAM), which provides three-dimensional (3D) microscopic images of optical absorbers in living tissues, was first proposed by Wang^1^^,^^2^ and has been extensively studied over the past 15 years.^3^^,^^4^ OR-PAM is highly sensitive to hemoglobin and melanin, which are major intrinsic optical absorbers in the visible wavelength range. OR-PAM, which has a spatial resolution determined by the optical diffraction limit, has made it possible to image capillaries that could not be imaged in the past, using photoacoustic imaging or acoustic-resolution photoacoustic microscopy.^1^ Furthermore, using OR-PAM images acquired at multiple excitation wavelengths, oxyhemoglobin and deoxyhemoglobin can be distinguished based on their absorption spectra.^5^^,^^6^ The oxygen saturation of blood within the capillaries can be calculated as the percentage of oxyhemoglobin in total hemoglobin. Microscale information on oxygen saturation has been used in various applications including brain function^6^ and tissue metabolism.^5^^,^^7^ This technique is called multispectral OR-PAM (MS-OR-PAM) and is used to distinguish other optical absorbers such as melanin and contrast agents from hemoglobin.^7^^,^^8^ One of the biggest challenges of OR-PAM is the imaging speed, with the first reported OR-PAM requiring approximately 10 min to image a 1 mm square area.^1^ This is because OR-PAM, which acquires the depth profile of the optical absorbers along the optical axis, requires a 2D raster scan of the optical axis over the entire imaging area to acquire a 3D image. To accelerate the imaging speed, OR-PAM systems using faster mechanical scanners, including voice coil scanners, have been discussed.^9^^,^^10^ Subsequently, a high-speed OR-PAM system with a fast-scan mirror and high-repetition-rate pulsed light source was developed.^11^^–^^18^ High-speed OR-PAM provides functional information on tissue dynamics with high spatial and temporal resolutions. The unique features of high-speed OR-PAM are useful for monitoring transient phenomena in biological tissues, such as the transient response of the brain to stimuli.^19^ Another issue with OR-PAM is the excitation wavelength tunability. To perform high-speed OR-PAM, a light source that generates single-mode beam-quality optical pulses with pulse energies of tens of nanojoules and pulse repetition frequencies above 100 kHz is required. However, only a few wavelength-tunable lasers satisfy all these requirements.^20^ Recently, some researchers have used wavelength conversion based on stimulated Raman scattering;^18^^,^^21^^–^^23^ however, the tunable wavelength range is limited, and wavelength tuning is discrete. Owing to this limitation, tunable lasers, such as optical parametric oscillators with low pulse repetition rates, are still used in the near-infrared (NIR) wavelength region, excluding the fundamental wavelengths of NIR lasers, such as Nd:YAG lasers.^24^ Functional NIR contrast agents targeting environments or molecules specific to diseases, such as cancer, have been widely developed for photoacoustic imaging. High-speed OR-PAM with NIR-wavelength excitation enables measurement of the dynamics of the microenvironment and disease-related molecules.

Circulating tumor cells (CTCs) are tumor cells that are released from primary tumor sites and circulate in the bloodstream. CTCs are good candidates as diagnostic biomarkers and early indicators of metastasis.^25^ Furthermore, patients with increased CTC counts showed more aggressive disease progression and shorter overall survival.^25^ Several groups have detected melanoma CTCs using photoacoustic measurements^26^^–^^29^ and high-speed OR-PAM.^30^^–^^32^ Because melanoma cells produce melanin with a broad absorption spectrum, OR-PAM at the fundamental wavelength of NIR lasers, such as 1064 nm, can detect melanoma cells with high sensitivity. Their results suggested an advantage of OR-PAM over existing CTC assays that use blood samples to avoid sampling errors. Furthermore, a theragnostic technique that uses high-speed OR-PAM to detect CTCs and irradiates them with a high-power laser was proposed. To apply high-speed OR-PAM-based CTC detection technology to tumor cells other than melanoma cells, a functional NIR contrast agent that stains CTCs and a high-speed OR-PAM that can detect stained CTCs are required. NIR contrast agents with an absorption peak around 800 nm have been extensively developed using various optical absorbers, including small organic molecule dyes^33^ and gold nanoparticles.^34^ In contrast, those with an absorption peak around 1064 nm are relatively few in number owing to the limited selection of optical absorbers, such as semiconductor nanoparticles.^24^ To detect various NIR contrast agents, OR-PAM with a tunable excitation wavelength within the NIR range is needed.

Recently, nanosecond pulsed supercontinuum (SC) light sources with sufficient pulse energy for OR-PAM applications have been developed.^35^^–^^40^ We reported an OR-PAM system that realizes MS-OR-PAM over a wide wavelength range, including the NIR region, using a nanosecond pulsed SC light source.^41^^,^^42^ In the OR-PAM system, excitation light pulses with specific wavelengths can be extracted using a bandpass filter for a broadband light pulse from the visible region to the NIR region (500 to 2200 nm) generated from an SC light source. We demonstrated chromatic aberration-free MS-OR-PAM over a wide wavelength range using a focusing mirror instead of an objective lens.^41^ However, the imaging speed of the OR-PAM system is limited because of slow mechanical scanning, and transmission-mode measurements, which place the imaging target between the focusing mirror and the PA sensor, are not suitable for *in vivo* imaging.

In this study, to enable the *in vivo* detection of CTCs stained by an NIR contrast agent, we developed a high-speed OR-PAM system that enables fast optical scanning and reflection mode measurement by arranging the focusing mirror and the PA sensor on the same side from the imaging target. The performance of the high-speed OR-PAM system in CTC detection was examined using a phantom experiment. Finally, we demonstrated the *in vivo* detection of CTCs using animal experiments.

## 2 Materials and Methods

### 2.1 Optical Resolution Photoacoustic Microscopy System Using Supercontinuum Light Source

**Fig. 1:**
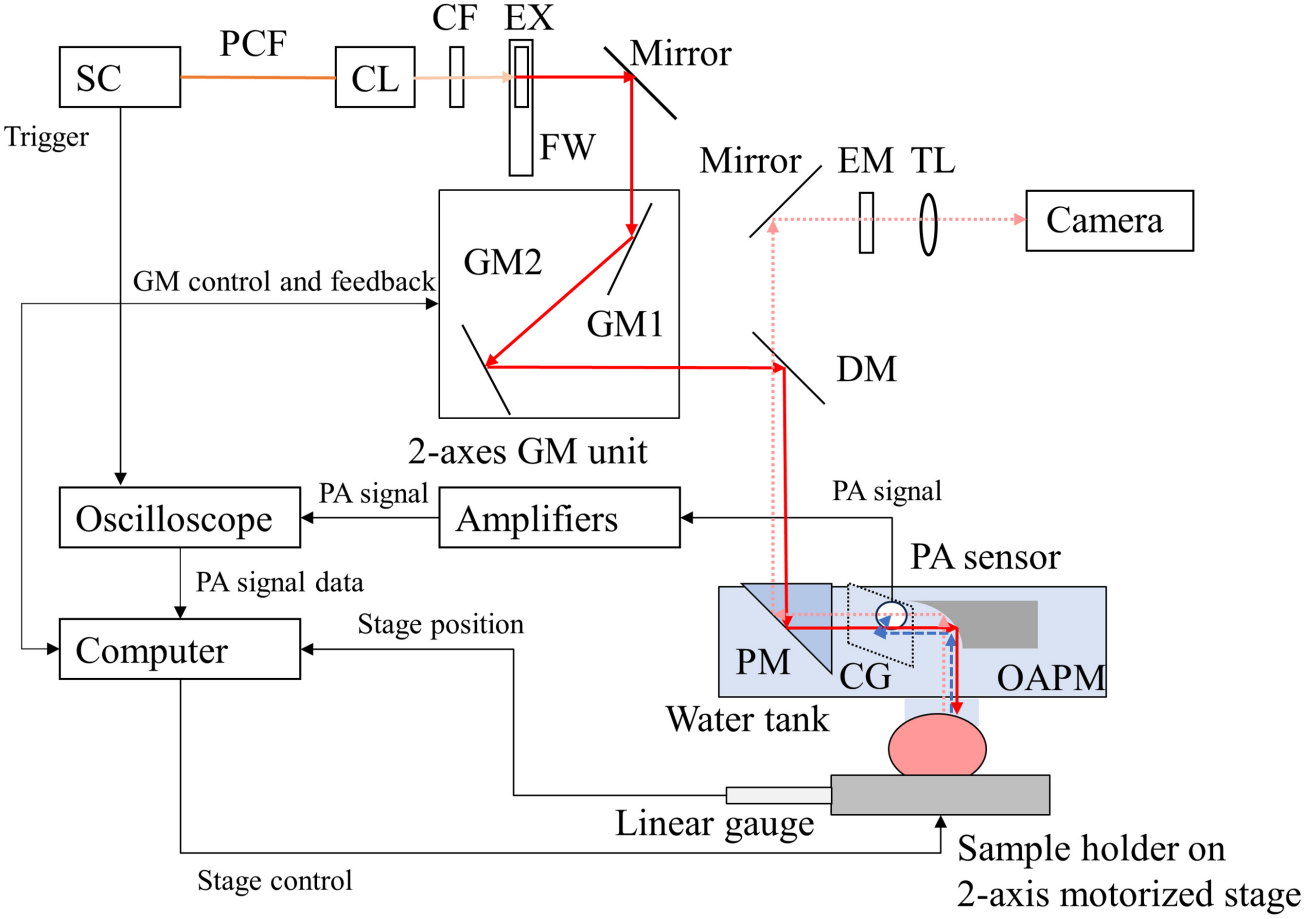
Schematic of a high-speed OR-PAM with SC light source. SC, supercontinuum; PCF, photonic crystal fiber; CL, collimator; CF, cold filter; FW, filter wheel; EX, excitation filter; DM, dichroic mirror; EM, emission filter; GM, Galvano mirror; PM, prism mirror; CG, cover glass; OAPM, off-axis parabolic mirror; and TL, tube lens.

Figure 1 shows a schematic of the high-speed OR-PAM system. The OR-PAM system was built by customizing a microscope system (CUS-BF; Sigma Koki, Tokyo, Japan). A nano-second pulsed light source (SC-Pro-HP, YSL Photonics, Wuhan, China) generated excitation light pulses with broadband wavelengths ranging from 500 to 2100 nm at a repetition rate of 100 kHz. The excitation light pulses were filtered using a cold filter (SC1101, Asahi Spectra, Tokyo, Japan; cutoff wavelength = 1100 nm) and a bandpass filter mounted on a filter wheel (FW1A, Thorlabs, Newton, New Jersey, United States) to select the excitation wavelengths. The pass bands of the bandpass filter used for hemoglobin imaging were 550 to 600 nm (#86-952, Edmund Optics, Barrington, New Jersey, United States), and those for NIR contrast agent imaging were 748 to 789 nm (#84-105, Edmund Optics, Barrington, New Jersey, United States). The excitation light pulses were reflected by a two-axis Galvano mirror (GCM012/M, Thorlabs, Newton, New Jersey, United States), a dichroic mirror (#86-336, Edmund Optics, Barrington, New Jersey, United States), and a right-angle prism mirror (MRA10-F01, Thorlabs, Newton, New Jersey, United States). Instead of an objective lens, an off-axis parabolic mirror (OAPM) (#37-289, Edmund Optics, Barrington, New Jersey, United States) with an effective focal length of 12.7 mm was used to focus the excitation light pulses. The OAPM is suitable for multispectral imaging over a wide range of wavelengths because the focal length of the reflective optics containing the OAPM is wavelength-independent. The focused excitation light pulse was irradiated onto an imaging target placed on a sample holder on a two-axis motorized stage (X: HPS80-50X-M5; Y and Z: SGSP-13ACTB0, Sigma Koki, Tokyo, Japan), and the optical absorbers in the imaging target at the focal point absorbed the excitation light pulses and produced PA signals. The off-axis parabolic mirror collimated the PA signals with spherical wavefronts and reflected them along the optical axis. A thin cover glass that transmitted light and reflected the ultrasound was placed on the optical axis at an incident angle of 45 deg to separate the PA signal from the optical axis. The PA signal was detected using a PA sensor made of a P(VDF-TrFE) piezoelectric copolymer film (KF-2#20, Kureha, Tokyo, Japan) with a peak frequency of 13.5 MHz, which was also immersed in a water bath. The PA sensor had a 6.0-mm-diameter circular detector element with a flat detection surface suitable for detecting collimated PA signals with plane wavefronts. For the PA signal transmission from the imaging target to the PA sensor, the right-angle prism mirror, OAPM, and PA sensor were immersed in a water bath. The bottom of the water bath was partly replaced by a 11-μm-thick clear film, and the film was contacted to the imaging target through a clear ultrasound coupling gel (F Jelly Plus Ultrasound Gel Soft, FUJIFILM, Tokyo, Japan). The top surface of the right-angle prism mirror was placed above the water surface to avoid the refraction of the excitation light pulses on the water surface. A pair of amplifiers (SA-220F5 and SA-230F5; NF Corporation, Kanagawa, Japan) and an attenuator (R411820124; Radiall, Tempe, Arizona, United States) amplified the PA signal with gain of 72 dB. The PA signals were filtered using a low-pass filter (EF526, Thorlabs, Newton, New Jersey, United States) with a cutoff frequency of 15 MHz, and then they were measured using a digital oscilloscope (PXIe-5164, National Instruments, Austin, Texas, United States) operating at a sampling frequency of 500 MHz. The depth profiles of the optical absorbers along the optical axis were calculated as envelopes of the temporal waveforms of the PA signals. Three-dimensional images were acquired by raster-scanning the optical focus over the imaging area. Thus, OR-PAM can perform both wide-field mechanical and fast optical scans. In wide-field mechanical scanning, raster scanning is performed by mechanically translating an imaging target using a two-axis motorized stage. An x-z cross-sectional image (B-scan image) was acquired by measuring the PA signals while translating the imaging target along the x-axis. A 3D image was obtained by acquiring the B-scan images while translating the imaging target along the y-axis. Acceleration and deceleration times of 50 ms were applied to reduce the inertia and maximize the scanning speed of the motorized stage. In this condition, the maximum imaging speed was 25  mm/s, which corresponds to a 4  μm pitch scan with 16 averages with a 100 kHz repetition rate excitation light pulse. To compensate for stage speed fluctuations, the motorized stage position was recorded using a linear gauge (LGK-0110, Mitutoyo Corporation, Kanagawa, Japan) and an analog counter (ELVIS II+, National Instruments, Austin, Texas, United States) synchronized with a digital oscilloscope. The imaging area of the wide-field scanning was limited by only the scan range of the motorized stages; whereas, its speed was limited by the scan speed of the motorized stage, acceleration and deceleration time, and communication between the motorized stages and a computer.

In fast optical scanning, the optical focus is scanned by steering the excitation light pulse using a two-axis Galvano mirror. An analog I/O device (ELVIS II+, National Instruments, Austin, Texas, United States) synchronized with a digital oscilloscope sent the control signal to and received the feedback signal from the two-axis galvano-mirror. The maximum scan area of the fast optical scanning, limited by the sensitive volume of the PA sensor, was 200  μm square. The maximum B-scan rate, limited by the operating frequency of the two-axis galvano-mirror, exceeded 300 fps. All instruments were controlled using LabVIEW software. Image reconstruction and signal analysis were performed using a laboratory-developed MATLAB program.

The OR-PAM system could detect the fluorescence emitted from an NIR contrast agent in an imaging target irradiated by an NIR PA excitation light pulse. Fluorescence was passed through a dichroic mirror (#86-336, Edmund Optics, Barrington, New Jersey, United States; cut-off wavelength = 801 nm), and an emission filter (#84-107, Edmund Optics, Barrington, New Jersey, United States; pass band = 814 to 851 nm) was focused using a tube lens (CU-040, SigmaKoki, Tokyo, Japan; focal length = 200 mm) and then detected using a monochrome charge-coupled diode camera (DMK33G618, The Imaging Source, Charlotte, North Carolina, United States).

### 2.2 Imaging of the Test Target

The imaging performance of the OR-PAM system was validated by imaging the USAF 1951 test target (R1DS1P; Thorlabs, Newton, New Jersey, United States). The wavelength range of the excitation light was 550 to 600 nm, and the pulse energy was 3  nJ/pulse. A wide-field image was acquired by scanning the test target within an area of $3.2\times 3.2\;{\mathrm{mm}}^{2}$ in 4  μm steps. To compare the mechanical scanning and the optical scanning, the test target was imaged in 1  μm steps within an area of $160\times 80\;\mu m^{2}$ by scanning both techniques. The PA signals were averaged 64 times to reduce random noise. To evaluate the spatial resolution of the OR-PAM system, an edge of the test target was imaged in 1  μm steps within an area of $100\times 100\;\mu m^{2}$ by mechanical scanning. The PA signals were averaged 256 times to reduce random noise. The full width at half maximum was calculated to measure spatial resolution by fitting the signal intensities along the x-axis to an edge-spread function.

### 2.3 Phantom Experiment

**Fig. 2:**
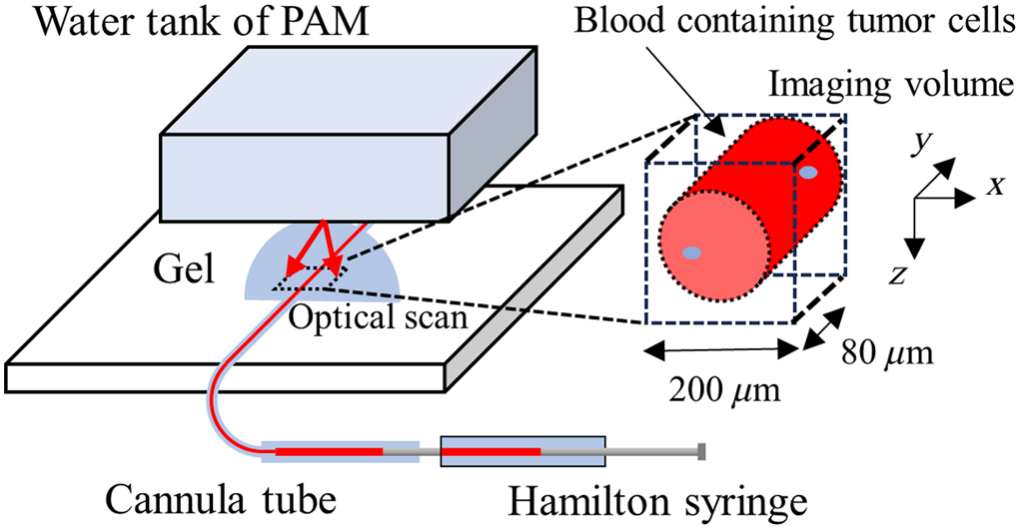
Experimental setup for the phantom experiment. OR-PAM image of blood-containing tumor cells in a cannula tube was sequentially acquired. The blood flowed at an average flow rate of 80  nL/s using a Hamilton syringe.

The performance of the OR-PAM system for CTC detection was examined through a phantom experiment. Prostate cancer cells (PC-3) were grown in Dulbecco’s modified Eagle’s medium (DMEM) containing D-glucose, L-glutamine, and sodium pyruvate (11885–084; Life Technologies, Carlsbad, California, United States) supplemented with 10% fetal bovine serum (FBS, SH3091003; Life Technologies, Carlsbad, California, United States)) and 1% antibiotic-antimycotic (15240–062; Life Technologies, Carlsbad, California, United States) in 5% CO2. The cells were then poured into four centrifuge tubes and centrifuged at 300 × G for 4 min. The supernatants were removed from the tubes, and the cells were then resuspended in DMEM containing 100  μM of cell-staining dyes (CellBrite NIR750, Biotium, Fremont, California, United States). The suspended cells were incubated for 24 h with continuous mixing using a see-saw shaker (NA-M101; Nissin Rika, Tokyo, Japan), washed twice by repeated centrifugation at 300 × G for 2 min, and resuspended in DMEM. Cell suspensions at five different concentrations were prepared by dividing the cell suspension into five tubes and adding DMEM. Blood samples were collected from nude mice (BALB/c Slc-nu/nu, Japan SLC, Shizuoka, Japan) using a procedure approved by the National Defense Medical College Committee for Animal Use (approval number: 21040). Approximately 10  U/mL of heparin was added to the blood samples and mixed with the cell suspensions at a volume ratio of 3:1. The final concentrations of the stained cells in the blood samples were $6.3\times 10^{2}$, $1.6\times 10^{2}$, $3.9.\times 10^{1}$, $9.8\times 10^{0}$, and 0  cells/μL. Blood samples were injected into a 180-μm-inner-diameter cannula tube (C10PU-RFA1310, INSTECH, Plymouth Meeting, Pennsylvania, United States) using a Hamilton syringe (1702N, Hamilton, Reno, Nevada, United States). The setup of the phantom experiment is shown in Fig. 2. The cannula tube was attached to the OR-PAM sample holder and brought into contact with the bottom of the water tank using an ultrasound coupling gel. Blood samples in the tube were continuously imaged by optically scanning an imaging area of $200\times 80\;\mu m^{2}$ in steps of 2.5  μm in the x direction and 2.0  μm in the y direction. For each concentration, 301 volumetric images of the blood samples were sequentially acquired at a volumetric imaging rate of 7.53 fps while the blood sample flowed at an average rate of 80  nL/s. The excitation wavelength and pulse energy were 748 to 789 nm and 133 nJ per pulse, respectively. OR-PAM images were acquired for the excitation wavelength range and pulse energies of 550 to 600 nm and 70  nJ/pulse, respectively, as hemoglobin contrast images. This experiment was repeated four times for each concentration.

Maximum amplitude projection (MAP) images along z-axis were calculated, a spatial averaging along x-axis (±2  pixels) was performed on the MAP images, and then the maximum signal intensity and the average signal intensity of the MAP images were calculated. Image frames in which the stained cells were visualized were detected by thresholding the maximum signal intensity by the mean + 4 SD of the maximum signal intensity in the control (0  cells/μL). The cell-detection rate was calculated by dividing the number of detected frames by the total number of frames. The first 40 frames and last 10 of the 301 frames were excluded from the analysis because of unstable flow rates.

For comparison with the measurements, the cell detection rate was calculated for each cell concentration. Because the tube was set parallel to the y-direction of scanning, volume of the blood sample can be calculated as $V=\pi (D/2{)}^{2}Y$, where D is inner diameter of the tube (180  μm) and Y is scanning range in the y-direction (80  μm). The average number of cells in the imaging volume was calculated by multiplying the volume (2.0 nL) by the cell concentration. The cell detection rate P was calculated as $P=1-e^{-p}$ where p is averaged number of cells in the imaging volume. The calculated cell detection rate indicated the percentage of image frames in which one or more tumor cells were detected.

### 2.4 *In Vivo* Imaging of Circulating Tumor Cells

*In vivo* detection of stained tumor cells within blood vessels was performed using procedures approved by the National Defense Medical College Animal Use Committee (approval number 21040). Prostate cancer cells (PC-3) were stained as described in Sec. 2.3 and then suspended in DMEM at concentrations of $2.5\times 10^{3}$, $5.0\times 10^{3}$, and $1.0\times 10^{4}\text{ cells}/\mu L$. Male nude mice (BALB/c Slc-nu/nu, Japan SLC, Shizuoka, Japan) were anesthetized by intraperitoneal injection of ketamine (90  mg/kg) and xylazine (9  mg/kg). Photographs of the experimental setup are shown in Fig. S4 in the Supplementary Material. The mouse was placed on the sample holder of the OR-PAM system, and the ear was fixed on the sample holder. The ear was placed in contact with the bottom of an OR-PAM water tank using an ultrasound coupling gel. First, a wide-field mechanical scan was performed with an imaging area of $3200\times 3200\;{\mu m}^{2}$ in 4  μm steps to acquire the vascular pattern of the ear. Next, a fast optical scan was performed with an imaging area of $160\times 80\;{\mu m}^{2}$ in 2  μm steps to confirm the presence of vascular within the imaging area. Vascular images were acquired with an excitation wavelength range of 550−600  nm and pulse energies of 70  nJ/pulse. Sequential OR-PAM imaging was then performed with an excitation wavelength range of 748 to 789 nm and a pulse energy of 133  nJ/pulse to detect the stained tumor cells. In sequential OR-PAM imaging, a series of high-speed optical scans was performed over the same imaging region. Three-thousand volumetric images were continuously acquired at a volumetric imaging rate of 7.53 fps. Immediately after starting the sequential OR-PAM imaging, 100  μL of the cell suspension was injected in tail vein of the mouse.

MAP images along z-axis were calculated, a spatial averaging along x-axis (±2  pixels) was performed on the MAP images, and then the maximum pixel value and the average pixel value of the MAP images were calculated.

## 3 Results and Discussions

### 3.1 Imaging of the Test Target

**Fig. 3:**
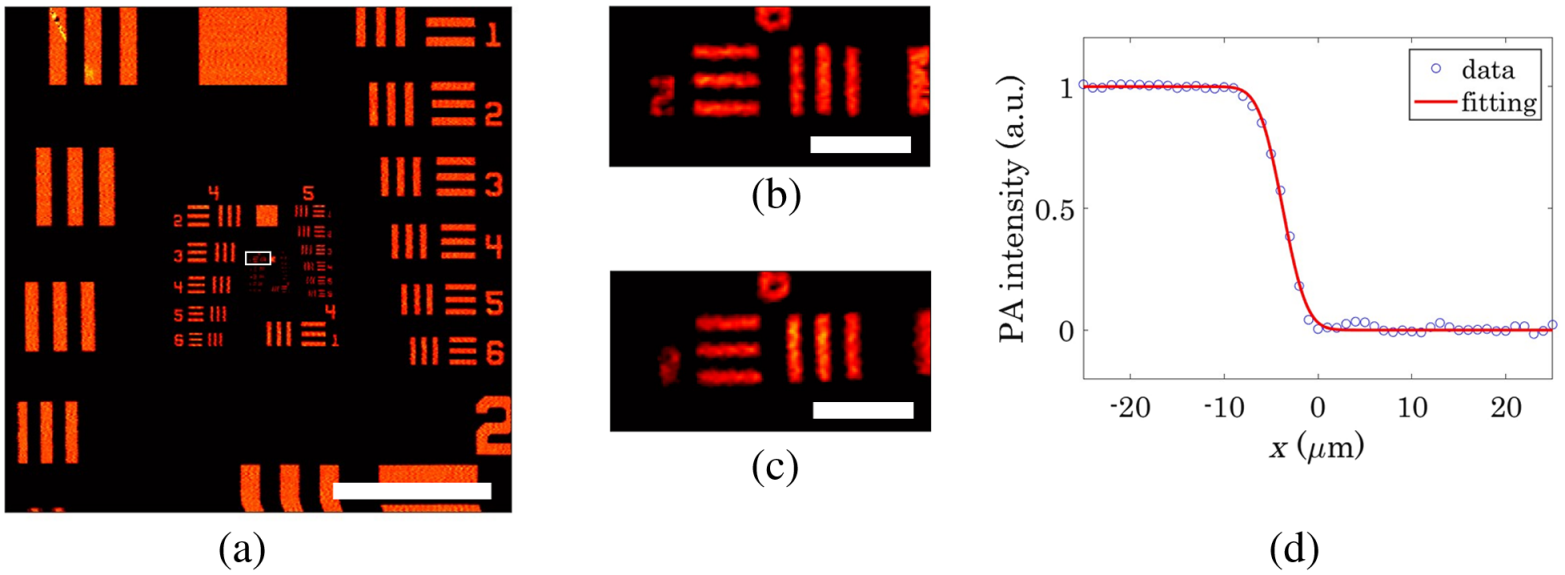
OR-PAM images of the test target. (a) Wide-field image acquired by the mechanical scan; the white rectangle on the image indicates the image area of (b) and (c), scale bar is 1 mm. Magnified images acquired by the mechanical scan (b) and optical scan (c), and scale bars are 50  μm. (d) PA signal intensity profile on the edge of the test target pattern fitted to an edge spread function.

The MAP of the OR-PAM image of the test target acquired by mechanical scanning is shown in Fig. 3(a). Mechanical scanning can be used to image the entire pattern of the test target. The MAP of the OR-PAM images acquired via mechanical and optical scanning are compared in Figs. 3(b) and 3(c), respectively. The imaging area is represented by the white rectangle in Fig. 3(a). The image quality of optical scanning was comparable to that of mechanical scanning. The spatial resolution of OR-PAM was measured by fitting the edge pattern to an edge-spread function, as shown in Fig. 3(d). The spatial resolution of 3.80±0.39  μm was determined from the fitting results of 81 scan lines. The spatial resolution was almost comparable to the diffraction-limited spot size of the optical focus (4.01  μm) was calculated as 0.51λ/NA [2,40], where NA is the numerical aperture. A numerical aperture of 0.073 was calculated from the beam diameter of the OAPM input (1.86 mm) and focal length (12.7 mm).

### 3.2 Phantom Experiment

**Fig. 4:**
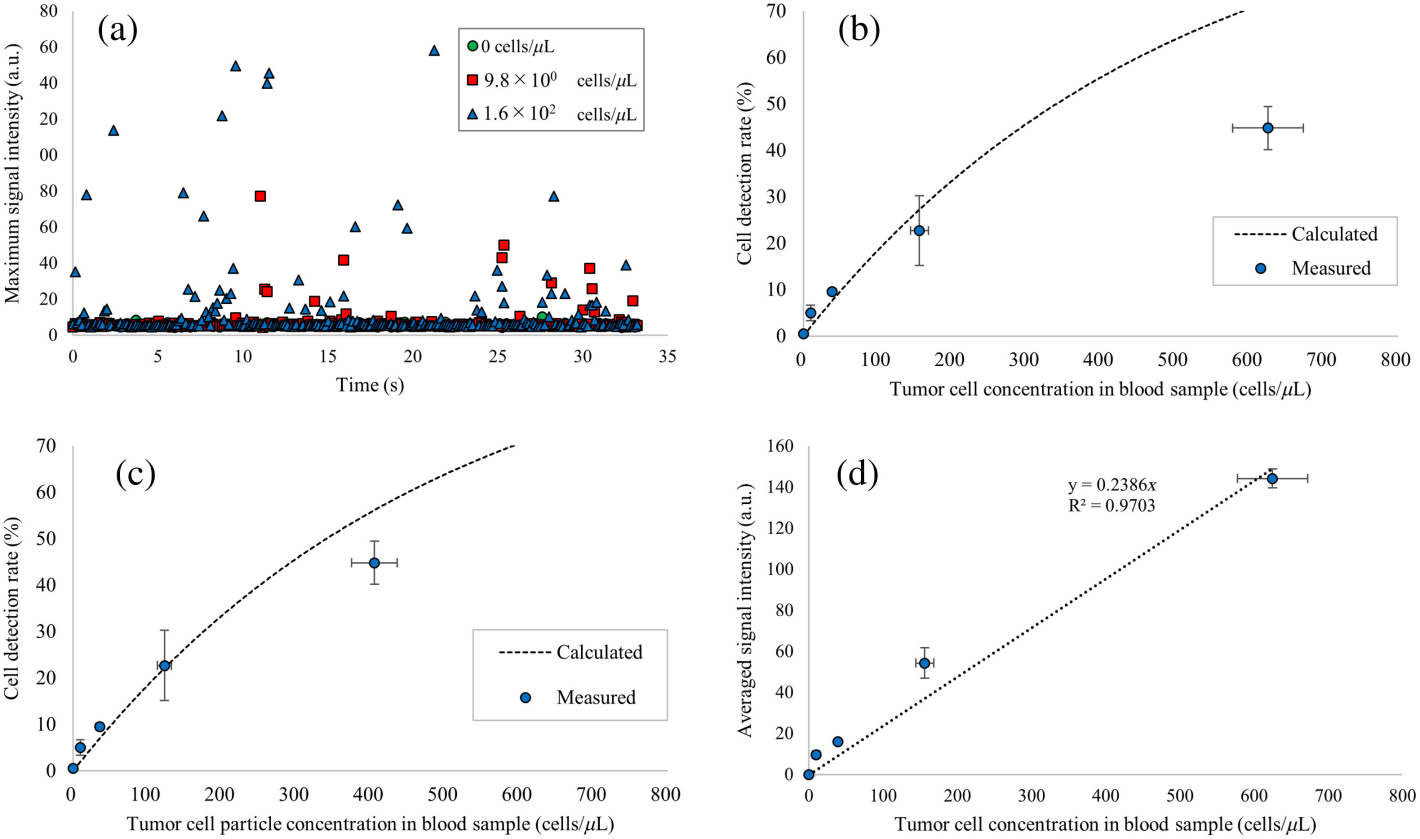
Result of the phantom experiment. (a) Time-course of maximum PA signal intensities from blood samples containing tumor cells at concentrations of 0 (green circle), $9.8\times 10^{0}$ (red square), and $1.6\times 10^{2}$ (blue triangle) cells/μL. Relation of the cell concentration in blood samples and cell detection rate (b) before correction and (c) after correction. (d) Relation of cell concentration in a blood sample and averaged PA signal intensity.

The time course of the maximum signal intensity within the imaging volume is shown in Fig. 4(a). Signal peaks were observed more frequently in the blood samples containing high concentrations of tumor cells. The relationship between the tumor cell concentration in the blood sample and the cell detection rate is shown in Fig. 4(b). The measured cell detection rate increased with increasing tumor cell concentration in blood samples. The cell detection rate was not proportional to the tumor cell concentration in the blood sample, because multiple cells could be detected within the imaging volume in the high-concentration range. The measured cell detection rate was comparable to the calculated cell detection rate in the low concentration range but deviated significantly from the calculated cell detection rate in the high concentration range. This deviation is attributed to a decrease in the number of cell particles resulting from the formation of cell clusters. The maximum signal intensity displayed in Fig. 4(a) reflects the PA signal intensity from a single particle within the imaging volume; thus, the average value of the maximum signal intensity should be constant for well-dispersed cells. However, as shown in Fig. 4(a), the average value of the maximum signal intensity was higher at higher cell concentrations. Thus, in the high concentration range, the average number of tumor cells in a single particle increased owing to cell cluster formation. To compensate for this, the tumor cell particle concentration was calculated by dividing the tumor cell concentration by a cluster factor. Cluster factor CF for each cell concentration c was calculated as $\mathrm{CF}(c)=S_{\max}^{\mathrm{ave}}(c)/S_{\max}^{\mathrm{ave}}(c_{\min})$, where $S_{\max}^{\mathrm{ave}}(c)$ is the average value of the maximum signal intensity at concentration c, and $S_{\max}^{\mathrm{ave}}(c_{\min})$ is the average value of the maximum signal intensity at the lowest concentration. The relationship between the tumor cell particle concentration in the blood sample and the cell detection rate was shown in Fig. 4(c). The measured cell detection rates closely matched the calculated cell detection rates.

We also analyzed the relationship between tumor cell concentration and average signal intensity in the imaging volume. As shown in Fig. 4(d), the average signal intensity was almost proportional to the tumor cell concentration. This result indicates that the total number of detected cells was proportional to the cell concentration.

### 3.3 In Vivo Imaging of Circulating Tumor Cells

**Fig. 5:**
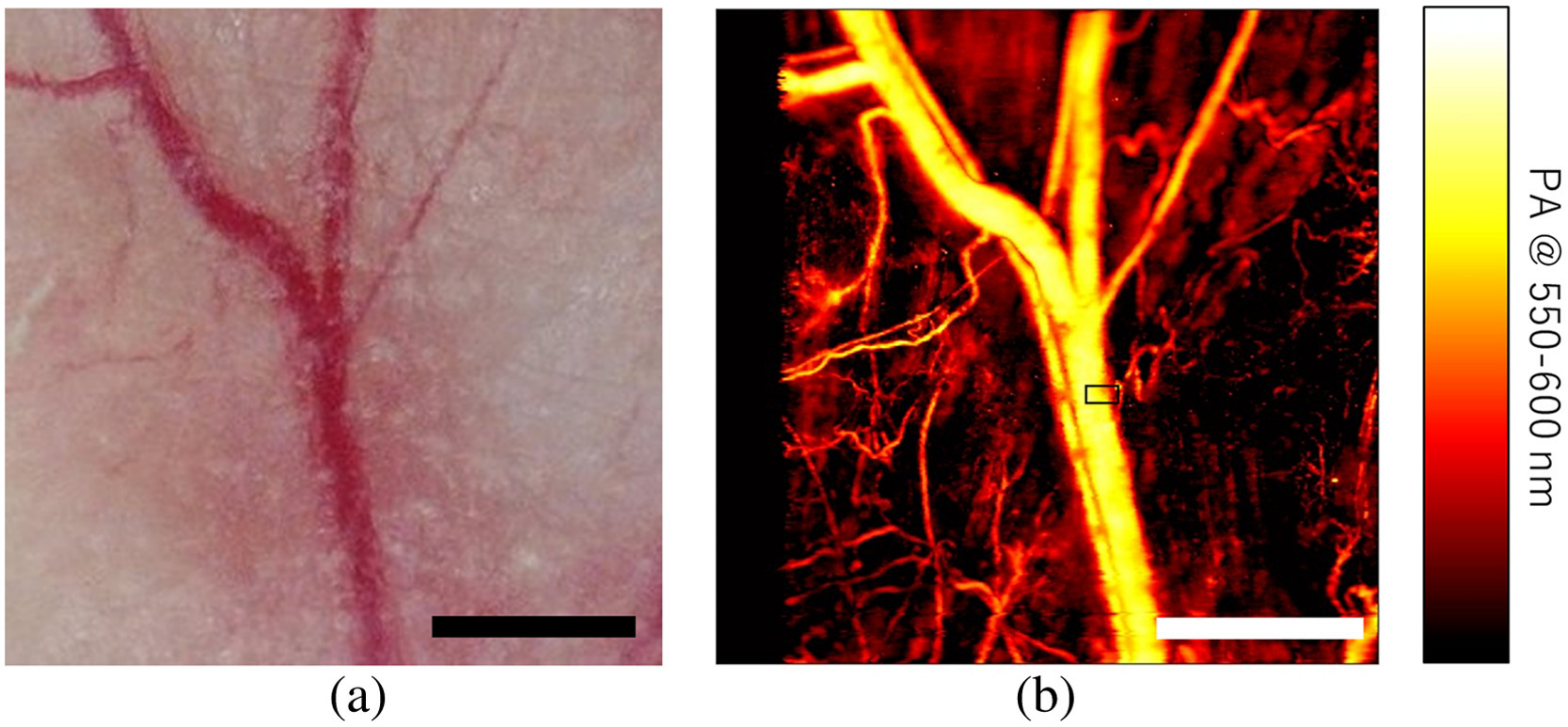
*In vivo* wide-field OR-PAM imaging of a mouse ear. (a) Optical microscopy image and (b) OR-PAM image. Scale bars are 1 mm, and black rectangle on panel (b) indicates imaging the region of fast optical scanning.

A wide-field OR-PAM image of the mouse ear acquired at an excitation wavelength range of 550 to 600 nm is shown in Fig. 5(b). Compared with the optical microscopy image shown in Fig. 5(a), the thin blood vessels and relatively thick blood vessels were clearly imaged by OR-PAM. To enhance the probability of tumor cell detection by maximizing the imaging volume, we chose blood vessels as the imaging area for detecting tumor cells in the blood.

**Fig. 6:**
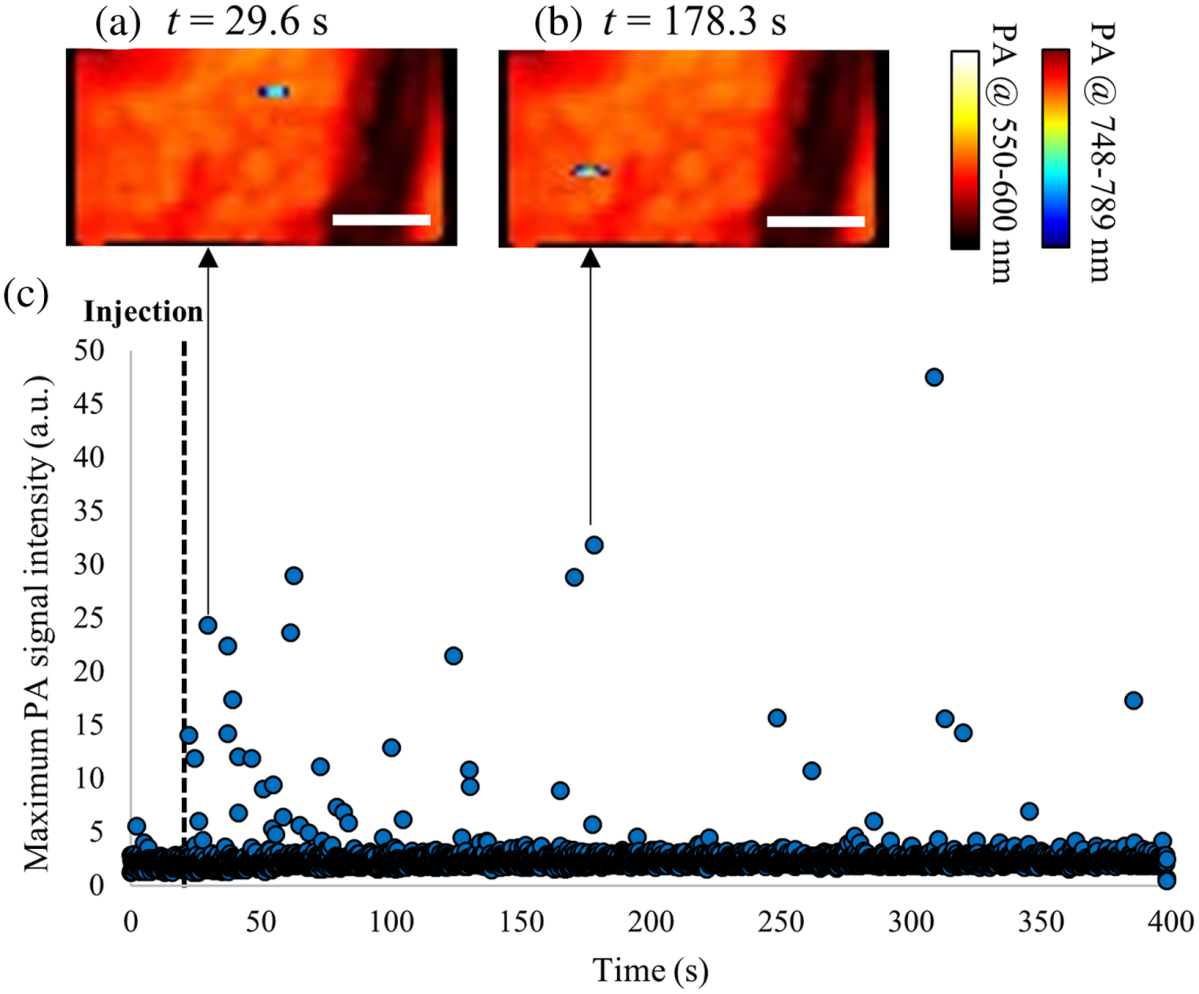
*In vivo* sequential OR-PAM imaging of a mouse ear during tail-vein injection of tumor cells. (a), (b) Multispectral OR-PAM images acquired at 550 to 600 nm excitation (hemoglobin contrast) and 748 to 789 nm excitation (stained tumor cells contrast) and (c) time-course of the maximum PA signal intensity at 748 to 789 nm excitation. The scale bar indicates 40  μm.

The results of sequential OR-PAM imaging to detect stained tumor cells are shown in Fig. 6. The time course of the maximum pixel values within the imaging area is shown in Fig. 6(a). Because of the low absorption coefficient of hemoglobin at NIR wavelengths, only weak PA signals were detected in the blood vessels before injection of the tumor cells. As shown in Fig. 6(a), signal peaks were detected after tail vein injection of stained tumor cells. In both the OR-PAM images shown in Figs. 6(b) and 6(c), the detected tumor cells were present in the blood vessels. This result demonstrates the possibility of *in vivo* tumor cell detection using the OR-PAM system.

**Fig. 7:**
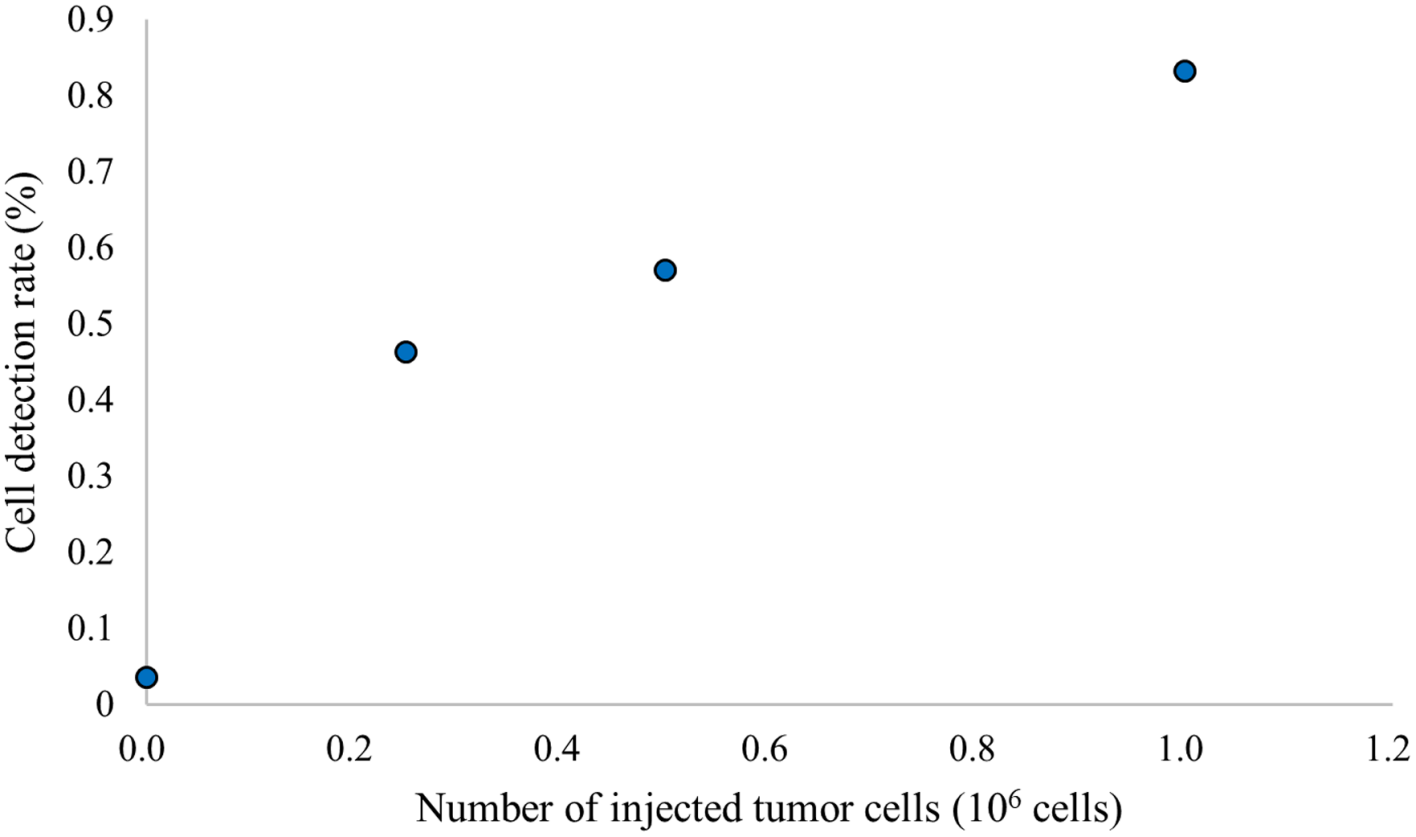
Relationship between the number of injected tumor cells and cell detection rate. (n=3 for $1.0\times 10^{6}\text{ cells}$, n=1 otherwise).

Figure 7 shows the relationship between the number of injected tumor cells and cell detection rate. The cell detection rate decreased with a decrease in the number of injected tumor cells. This indicates that the tumor cell detection rate reflects the tumor cell concentration in the blood.

A limitation of *in vivo* experiments is that the tumor cell concentration in the ear blood vessels is different from the injected tumor cell concentration because there is non-negligible tumor cell entrapment within the pulmonary capillaries. *In vivo* whole-body fluorescence image obtained after injection of the tumor cells showing lung accumulation of the cells is shown in Fig. S5 in the Supplementary Material. Therefore, unlike phantom experiments, directly comparing tumor cell concentration in blood and tumor cell detection rate using the OR-PAM was difficult. However, as shown in Fig. 7, the tumor cell detection rate decreased with a decrease in the injected tumor cell concentration.

Although we have demonstrated that the OR-PAM system can detect stained tumor cells within blood vessels, three challenges remain for practical application. The first challenge is the throughput of blood sampling. The number of CTCs in the blood sampled from post-therapy patients has been suggested as a biomarker for prostate cancer prognosis. Patients with CTCs counts of 5 or more in 7.5 mL of blood at 4 weeks after therapy showed significantly shorter overall survival.^43^ CTC detection technology using OR-PAM shows good sensitivity for low-concentration CTC, and noninvasive continuous measurement is possible; therefore, it is expected to achieve sufficient sensitivity for long-term continuous measurements. In our experimental conditions, the imaging volume per single image was 9 nL, assuming an imaging depth of 0.7 mm. Multiplying by the frame rate of 7.53 fps, the OR-PAM blood sample throughput is calculated to be 67.4  nL/s. Therefore, approximately 31 h is required to sample 7.5 mL of blood. The throughput can be improved by operating the light source at a higher repetition rate, reducing the number of signal averaging, and increasing the sampling pitch. For instance, by operating the light source at its maximum repetition rate of 1 MHz, acquiring signals without averaging, and increasing the scan pitch to 4  μm, the throughput can be improved by a factor of 160. Such high-speed scanning conditions are rendered possible using a faster scanner such as a polygon mirror.^13^ The second challenge is device miniaturization. Even with increased throughput, sampling 7.5 mL of blood requires continuous monitoring for more than 10 min. Therefore, miniaturized OR-PAM devices that are robust to motion are needed. Other groups have achieved miniaturization of the OR-PAM device, and one such group has developed a wearable device.^44^ The third challenge is *in vivo* cell staining. In this experiment, stained tumor cells were injected into blood vessels to test the performance of the OR-PAM system in detecting well-stained tumor cells. For clinical applications, tumor-leaked CTCs must be specifically stained within blood vessels. For this purpose, a molecular probe that can specifically stain tumor cells is required. Various molecular probes with various targeting ligands and optical absorbers (e.g., small organic molecule,^45^ metal nanoparticles,^34^ and carbon nanoparticles^46^) have been developed.^47^ Therefore, the next step is to determine a suitable molecular probe for CTC detection and use that molecular probe to detect CTCs.

## 4 Conclusion

In this study, we developed a high-speed OR-PAM equipped with an SC light source and a Galvano scanner, enabling real-time volumetric imaging with excellent wavelength tunability in the visible-to-NIR wavelength range. The application of the OR-PAM system for CTC detection was discussed. A phantom experiment was performed using blood samples containing stained tumor cells. The OR-PAM system successfully detected stained tumor cells, and the cell detection rate was correlated with the cell concentration in the blood sample. Furthermore, *in vivo* experiments to detect the stained tumor cells injected into the blood were performed in mouse ears. Stained cells were detected after injection of tumor cells. This result demonstrates the ability of PAM for *in vivo*. As a next step, we plan to combine the OR-PAM technology with molecular probes to stain CTCs *in vivo* and then detect CTCs using the OR-PAM system.

## Supplementary Material



## Data Availability

The data underlying the results presented in this paper are not publicly available at this time but may be obtained from the authors upon reasonable request.
