# Supplementary material for: Photoacoustic microscopy for real-time monitoring of near-infrared optical absorbers inside biological tissue
Source: J Biomed Opt. 2024 Mar 9;29(Suppl 1):S11527. doi: 10.1117/1.JBO.29.S1.S11527 (PMC10924425; doi:10.1117/1.JBO.29.S1.S11527)
Supplement: Supplementary file 1 [file JBO_029_S11527_SD001.pdf]

Supplementary information

Cell detection probability

Averaged number of cells within the detection volume is p. If dividing the detection volume into N small volumes, averaged number of cells in the small volumes becomes p/N. Thus, (1-p/N) is the probability that a small volume contains no cells. When all small volumes contain no cells, the detection volume contain no cells. Therefore, the probability that the detection volume contain no cells is $(1\text{-}p/N)^N$. By calculating limit of the probability as N approaches infinite, the probability that the detection volume contain no cells can be calculated as following.

$$\lim_{N\to\infty}\left(1-\frac{p}{N}\right)^N=\left\{\lim_{n\to-\infty}\left(1+\frac{1}{n}\right)^n\right\}^{-p}=e^{-p}$$

Thus, the probability that the detection volume contain at least one cell is $P=1\text{-}e^{-p}$.

Calculation of cluster factor (CF)

In section 3.2, we used cluster factor to compensate for decrease of cell particle number due to cell cluster formation. As shown in Fig. 4a, maximum signal intensity increased with increasing cell concentration. The increase of the maximum signal intensity suggested that the formation of cell clusters that produces stronger PA signals. To compensate for the effect of cell cluster formation, we defined cluster factor (CF) as averaged number of cells in a cell particle. Fig. S1 shows relation between the cell concentration and maximum signal intensity $s_{max}(c)$. Fig S2 shows cluster factor $CF(c) = S_{max}^{ave}(c)/S_{max}^{ave}(c_{min})$, where $S_{max}^{ave}(c)$ is the average value of the maximum signal intensity at concentration c, and $S_{max}^{ave}(c_{min})$ is the average value of the maximum signal intensity at the lowest concentration.

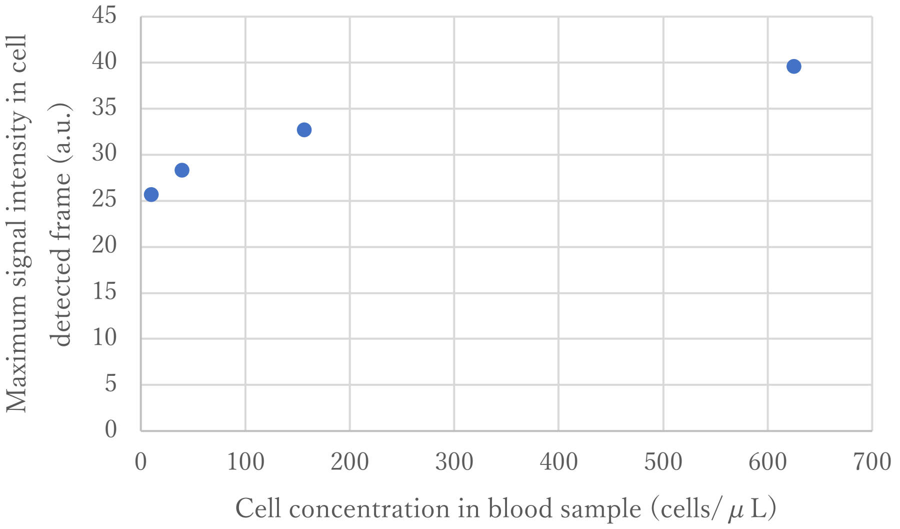

Fig S1 Relation between the cell concentration and maximum signal intensity.

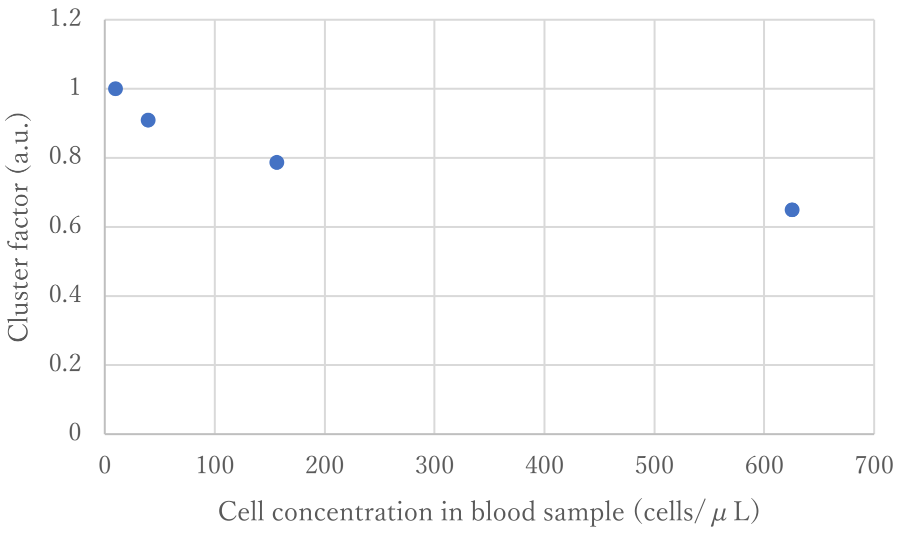

Fig S2 Relation between the cell concentration and cluster factor.

Comparison of OR-PAM and fluorescence measurement

In in vivo imaging of circulating tumor cells, fluorescence produced from the tumor cells was also measured. Fig S3 compares the cell detection rate of the OR-PAM (PA) and Fluorescence (FL). The cell detection rate gradually decreased over time in both PA and FL. The higher cell detection rate of FL immediately after injection may be due to expansion of fluorescence excitation beam.

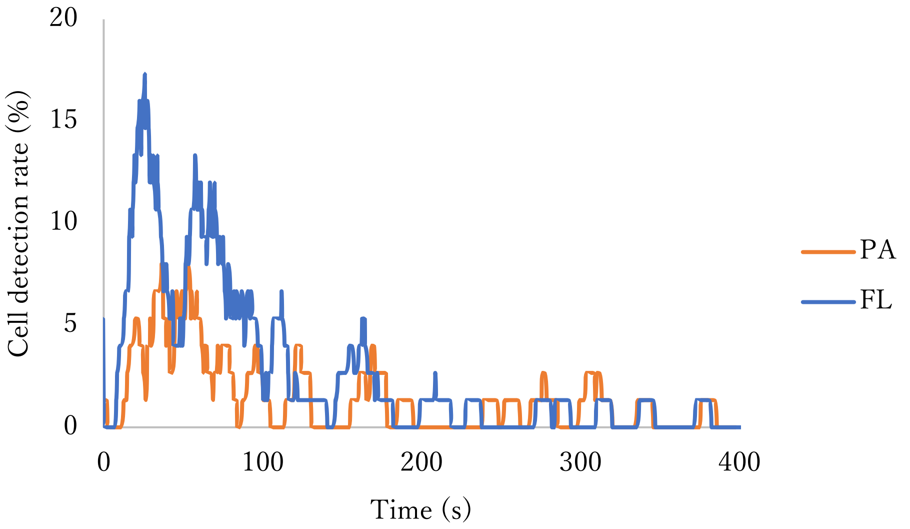

Fig. S3 Comparison of cell detection rate of the OR-PAM detection and the fluorescence detection. Cell detection rate was calculated as an average every 10 seconds.

Photographs of animal experiment setup

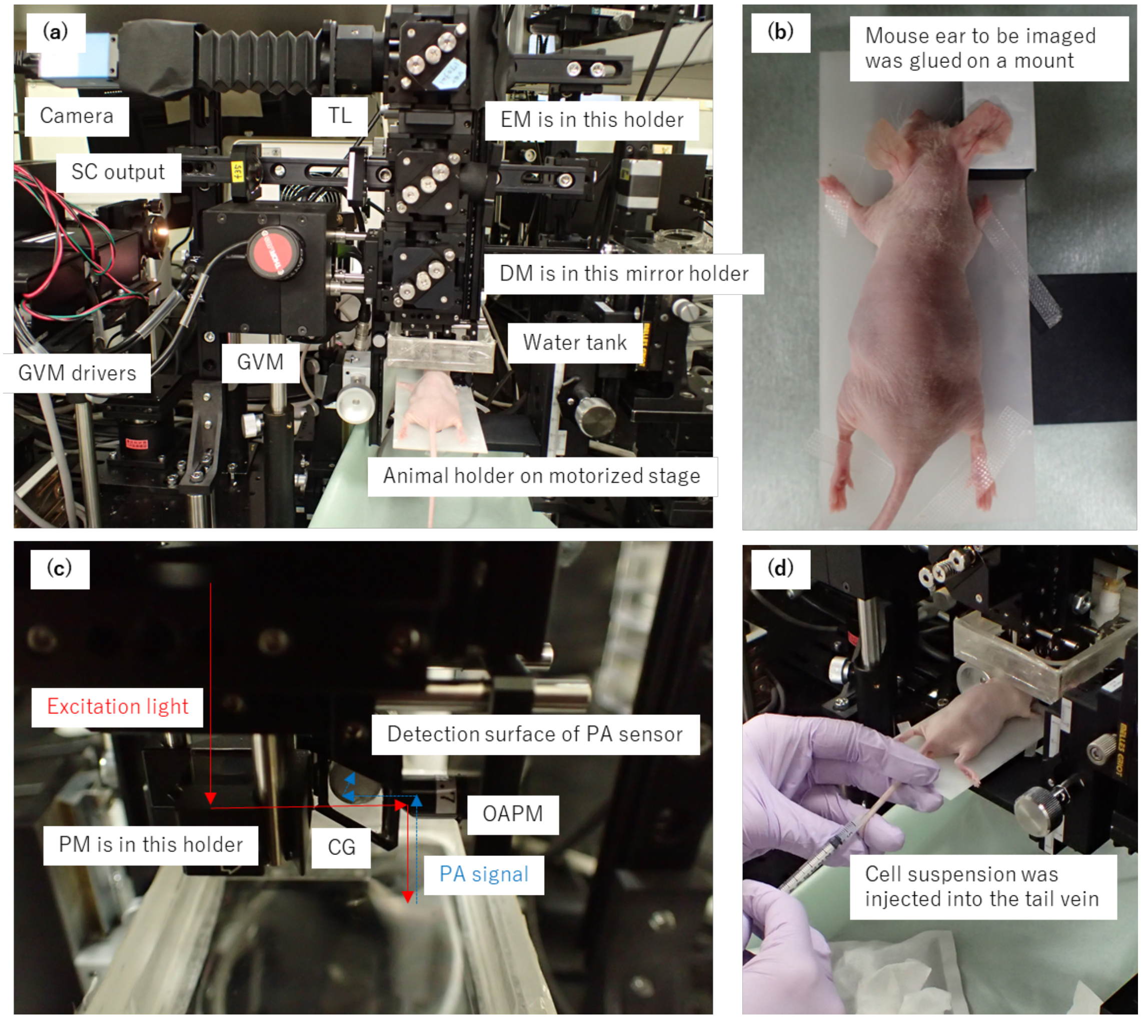

Fig. S4 Photographs explaining experimental setup for the animal experiment. (a) Overview of the PAM system. (b) The mouse on the animal holder. (c) Detection part of PAM system (this part is immersed in water tank during signal acquisition). (d) Injection of tumor cells into tail vein.

SC, Supercontinuum; DM, Dichroic mirror; EM, Emission filter; GVM, Galvano mirror; PM, Prism mirror; CG, Cover glass; OAPM, Off-axis parabolic mirror; TL, Tube lens.

Fluorescence image of a mouse after injection of tumor cells

Fluorescence image of a mouse after injection of tumor cells stained by CellBrite750 were captured by in vivo fluorescence imaging instrument (IVIS Lumina XRMS, PerkinElmer Inc., Waltham, MA, USA). Lung accumulation of tumor cells was observed from the fluorescence image.

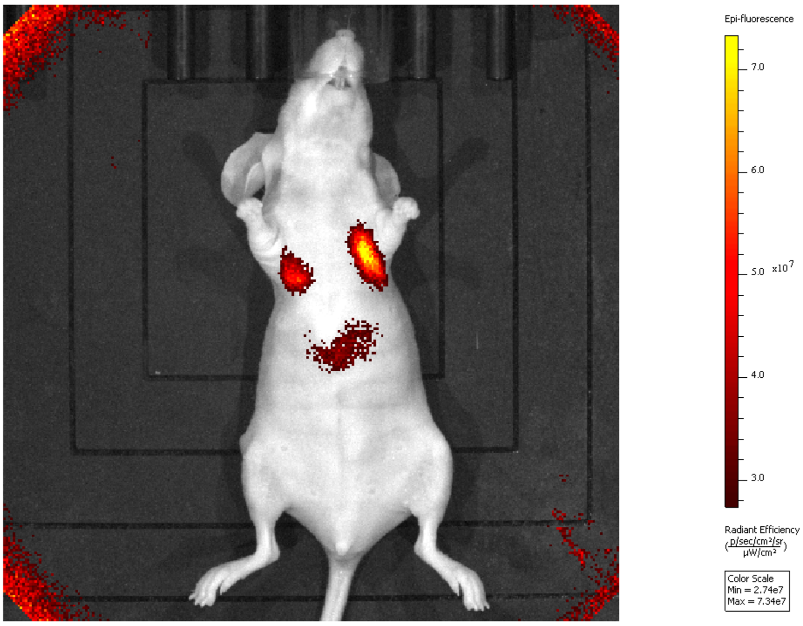

Fig. S5 In vivo whole-body fluorescence images obtained after injection of tumor cells.

Summary of imaging conditions

Table S1 Summary of imaging conditions

Target	Test target			Phantom	Mouse ear	
Scan method	Wide field	Fast optical		Fast optical	Wide field	Fast optical
Imaging area (X×Y, μm)	3200×3200	160 × 80	100 × 100	200 × 80	3200×3200	160 × 80
Number of scan points (X×Y)	801 × 801	161 × 81	101 × 101	81 × 41	801 × 801	81 × 41
Number of averaging	64	64	256	4	16	4
Imaging speed (s/frame)	820	8.35	26.1	1 / 7.53	820	1 / 7.53
Pulse energy (nJ @ center wavelength)	3 @ 575 nm	3 @ 575 nm	3 @ 575 nm	70 @ 575 nm<br>133 @ 769 nm	70 @ 575 nm	70 @ 575 nm<br>133 @ 769 nm
Figures	3a, 3b	3c	3d	4	5	6, 7
